# Sleep disturbance after pediatric and adolescent concussion

**DOI:** 10.3389/frsle.2025.1528458

**Published:** 2025-03-20

**Authors:** James Wilkes, Daniele Fedonni, Jelsia Cottone, Kristy B. Arbogast, Christina L. Master

**Affiliations:** ^1^Center for Injury Research and Prevention, The Children's Hospital of Philadelphia, Philadelphia, PA, United States; ^2^Department of Biomedical Health and Informatics, The Children's Hospital of Philadelphia, Philadelphia, PA, United States; ^3^Department of Pediatrics, Perelman School of Medicine, University of Pennsylvania, Philadelphia, PA, United States; ^4^Department of Orthopaedic Surgery, Perelman School of Medicine, University of Pennsylvania, Philadelphia, PA, United States; ^5^Sports Medicine and Performance Center, The Children's Hospital of Philadelphia, Philadelphia, PA, United States

**Keywords:** pediatric, adolescent, concussion, sleep, recovery

## Abstract

**Introduction:**

Sleep disturbances following concussion are common. The goal of this study was to describe subjective reports of sleep disturbances for pediatric and adolescent patients, whether they predict prolonged recovery or moderate the relationship between comorbidities, symptom burden, and recovery.

**Materials and methods:**

Clinical electronic health record (EHR) data from a prospective concussion registry of patients aged 5–18 were used for this study. Specific demographic and injury characteristics included sex, age, race and ethnicity, previous concussion history, symptom scores, and personal history of mental health diagnoses and sleep problems. Categorical variables were compared with Pearson's chi-squared tests and continuous variables were compared with Kruskal-Wallis rank sum test. Univariate and multivariate logistic regression were used to calculate odds ratios, 95% confidence intervals and *p*-values for factors associated with prolonged concussion recovery. Interaction terms were created for each comorbidity and sleep changes to test for moderation effects of comorbidities on the relationship between sleep disturbance and prolonged recovery.

**Results:**

A total of 4,469 patients with a concussion were seen within 28 days of injury during the study period and included in analyses, with 3,002 (67%) reporting new sleep disturbance. Those with sleep disturbance differed by sex, age, time from injury to initial visit, Post Concussion Symptom Inventory (PCSI) at initial visit, concussion history, presence of comorbidities, and COI. In the multivariate model, female patients (OR 1.43, 95% CI 1.25–1.64), those with new sleep disturbance (OR 1.37, 95% CI 1.18–1.60), patients without a previous concussion diagnosis (OR 1.31, 95% CI 1.15–1.52), medical history of a learning disability (OR 1.3, 95% CI 1.10–1.67), more days between injury to initial visit (CI 1.08–1.10), and a higher initial PCSI score (OR 1.03, 95% CI 1.03–1.03) had greater odds of prolonged recovery. Interaction terms for moderation effects of comorbidities on the relationship between sleep disturbance and prolonged recovery were not statistically significant.

**Discussion:**

Nearly 2/3 of concussion patients self-reported changes in sleep after injury, a higher rate than previously reported (51%). Sleep disturbances following concussion were the second strongest predictor of prolonged recovery past 28 days, only behind female sex, and comorbidities did not moderate that relationship.

## Introduction

Pediatric and adolescent concussion is a growing area of concern as 3.9% of all children in the US in 2021 have been diagnosed with a concussion sometime during childhood (Black and Zablotsky, 2021). Sleep disturbances with concussion are common, with up to 51% of pediatric and adolescent patients reporting increased sleep disturbance within 1 week following injury (Djukic et al., 2022). Both concussion and sleep disturbance in the absence of injury have been shown to lead to deficits in cognition, reaction time, perception, balance and proprioception, autonomic function, sleep-wake regulation, and mood regulation (Bauer and Jaffee, 2021; Zimmerman et al., 2024; Vandekerckhove and Wang, 2018). These deficits can broadly impact quality of life across several domains: academic, social, physical, emotional, and mental health. Particularly, sleep disturbance immediately following concussion is highly prevalent and predictive of persistent symptom burden (Bramley et al., 2017; Patricios et al., 2023), but has not been directly examined as it relates to clinical recovery in pediatric and adolescent patients.

A recent systematic review described five distinct concussion phenotypes—cognitive, ocular-motor, headache/migraine, vestibular, and anxiety/mood—with a concussion-associated condition of sleep disturbance affecting 33% of concussed individuals not specific to phenotype (Lumba-Brown et al., 2020). Few treatments exist to address this concussion-associated condition, making it crucial to advance understanding of post-injury sleep disturbances to improve clinical care. Thus, identifying the individual factors contributing to sleep disturbances after concussion is critical to informing prevention or mitigation tactics prior to injury, as well as effective treatments following injury.

While it is critical to consider individual demographic or injury factors that contribute to recovery, it is also paramount to include familial, environmental, and other external metrics that influence a patient's recovery pathway and potential. For example, recent pediatric research has identified the Child Opportunity Index (COI) as a useful composite measure of factors external to the child or family themselves that contribute to health and wellbeing. COI is a robust, nationally-normed score developed from census data as a composite measure of community-level infrastructure relating to resource availability and quality, as well as other geographic and neighborhood factors that contribute to healthy childhood development (COI 3.0 Overall Index and Three Domains, 2024). COI may be useful in identifying how these additional factors influence sleep disturbances after concussion.

It is also important to consider the intersection between sleep and mental health and other comorbidities identified as factors for increased risk for prolonged recovery after concussion. Specifically, poor sleep has been identified as a factor for worsening depression, anxiety, migraine/headache, ADHD, and other mental health conditions (Medic et al., 2017; Vgontzas and Pavlović, 2018; Scott et al., 2021; Charest and Grandner, 2020). Similarly, those with depression, anxiety, headache/migraine, etc. have been shown to have impaired sleep or disturbed circadian rhythm (Cox and Olatunji, 2016; Steiger and Pawlowski, 2019; Oh et al., 2019). Student-athletes specifically may be at higher risk for developing a bidirectional negative feedback loop (Sin et al., 2017) because of their high rates of sleep insufficiency, propensity for developing certain sleep disorders, and extrinsic and intrinsic pressures that lend to poor mental health (Charest and Grandner, 2020; Wang and Bíró, 2021; Kroshus et al., 2019). Insufficient sleep and poor mental health can further contribute to increased risk-taking behaviors that may lead to both increased risk for injury and impaired or prolonged recovery (Charest and Grandner, 2020). Within the context of concussion, individuals with pre-existing mental health conditions are at greater risk for prolonged recovery after injury (Master et al., 2024), but the contribution of sleep to this relationship is not well understood. Due to these overlapping symptoms and etiology, it is important to better understand how sleep relates to comorbidities of concussion to inform therapeutic techniques and clinical guidance during recovery.

The goal of this study was to leverage a large pediatric and adolescent concussion registry to describe subjective reports of sleep disturbances after injury, and to examine differences in these reports across demographic and clinical characteristics. A secondary goal of the study was to explore whether self-reported sleep disturbances after concussion predict prolonged recovery past 28 days within the same cohort. For recovery specifically, we hypothesized that those self-reporting sleep disturbances would have significantly higher risk for prolonged recovery past 28 days than peers not endorsing sleep disturbances. Finally, an additional exploratory goal of this study was to understand whether comorbidities moderated the relationship between disturbed sleep and prolonged recovery.

## Materials and methods

### Participants

Data were drawn from a prospective cohort of patients diagnosed with a concussion presenting to a specialty care concussion program between Jan 1, 2018, and June 4, 2024, from a concussion registry containing clinical electronic health record (EHR) data. Patients 5–18 years of age at initial visit, sustaining their concussion from all types of mechanisms of injury, were included in analyses. This study was approved by the Children's Hospital of Philadelphia Institutional Review Board.

### Procedures

Patients completed a standardized demographic questionnaire, which included sex, race, ethnicity, and age at visit. Additionally, patients reported previous medical history, including self-reported mental health diagnoses, such as anxiety, depression, attention deficit/hyperactivity disorder (ADD/ADHD), other leaning disability, bipolar disorder, other psychiatric diagnosis, and sleep problems, as well as concussion history. Concussion-related symptoms were evaluated using the validated Post-Concussion Symptom Inventory (PCSI; Sady et al., 2014). Patients 5–12 were used the PCSI-Child form and those 13–18 the PCSI-Teen form. PCSI-Child scores each symptom 0–2 while the PCSI-Teen scores each symptom 0–6. To directly compare PCSI scores across the two versions, each PCSI score was divided by the maximum item score then multiplied by 100 for a percentage of symptom severity as has been previously published (Corwin et al., 2024). The nationally-normed score for the overall COI, as well as the three COI subdomains (social/economic, educational, and health/environment), were generated based on the patient's address. COI scores range from 0 to 100, where higher scores represent a higher degree of opportunity. At initial visit, patients were asked if their sleep has been different compared to before injury. Patients who reported any changes to their sleep such as (trouble sleeping, sleeping more than usual, sleeping less than usual, frequently waking up, or difficulty waking up) were considered to have sleep-related disturbances. Finally, we defined prolonged concussion recovery as continuing to be in clinical care for the persistence of concussion symptoms >28 days after injury (Zemek et al., 2016).

### Statistical analyses

Standard descriptive statistics were used to describe the overall sociodemographic and clinical characteristics. Categorical variables were compared by reported sleep-related disturbances (no changes to sleep since injury and changes to sleep since injury) with Pearson's chi-squared tests while continuous variables were compared with Kruskal-Wallis rank sum test. Unadjusted and adjusted odds ratios, 95% confidence intervals, and *p*-values for sociodemographic and clinical characteristics associated with prolonged concussion recovery were calculated using univariate (unadjusted) and multivariate (adjusted) logistic regression. Factors associated with prolonged recovery (*p* < 0.05) in the univariate model were included in the multivariate regression analysis as potential cofounders. Log odds ratio, standard error, 95% confidence intervals and *p*-values were calculated for all factors that met the significance level of *p* < 0.05 in the univariate models. To test for moderation effects of comorbidities on the relationship between sleep disturbance and prolonged recovery, interaction terms were created for each comorbidity and sleep changes. Statistical analyses were performed using R 4.0.3 (R Foundation for Statistical Computing, Austria). Statistical level of significance was set *a priori* to 0.05.

## Results

### Demographic analysis

A total of 4,469 patients were diagnosed with a concussion and seen for a clinical visit within 28 days of injury during the study period, with 3,002 (67%) reporting sleep disturbance since injury at their initial visit to specialty care (Table 1). A significantly higher proportion of females reported changes to sleep (58%) compared to males (42%) (*p* < 0.001). The median age at initial visit was older among those who reported sleep changes (15 years) than those who did not (14 years) (*p* < 0.001). Additionally, patients experiencing changes to sleep had a longer median time from injury to initial visit (12 vs. 11 days, *p* < 0.001). PCSI also varied at initial visit with those reporting changes to sleep with a higher PCSI (37) compared to those who did not report changes (12) (*p* < 0.001).

**Table 1:** Selected characteristics of population cohort at initial visit.

**Characteristic**	***N***	***N* = 4,469^a^**
Age at initial visit	4,469	15.00 (13.00, 16.00)
Sex	4,469	
Female		2,377 (53%)
Male		2,092 (47%)
Race/ethnicity	4,469	
Hispanic		295 (6.6%)
Non-Hispanic black		521 (12%)
Non-Hispanic other		579 (13%)
Non-Hispanic white		3,074 (69%)
Days from injury to initial visit	4,469	12 (7, 19)
PCSI initial visit	4,469	29 (12, 47)
Previous concussion	4,469	2,804 (63%)
At least 1 of the selected comorbidities	4,469	1,960 (44%)
Medical history: anxiety	4,469	1,084 (24%)
Medical history: ADD/ADHD	4,469	771 (17%)
Medical history: learning disability	4,469	553 (12%)
Medical history: depression	4,469	625 (14%)
Medical history: bipolar disorder	4,469	56 (1.3%)
Medical history: other psychiatric disorder	4,469	138 (3.1%)
Medical history: chronic headache	4,469	434 (9.7%)
Medical history of sleep problems	4,469	523 (12%)
COI—overall	4,469	89 (70, 96)
COI—educational domain	4,469	90 (68, 96)
COI—health domain	4,469	84 (68, 93)
COI—social/environmental domain	4,469	86 (67, 95)

^a^Median (IQR); n (%).

Patients with a history of concussion less frequently (61%) reported sleep disturbance since injury than those with no previous concussion (66%) (*p* < 0.01).

Co-occurring medical conditions were also associated with higher percentage of patients reporting changes to sleep: anxiety (29% for those with anxiety vs. 15% with no anxiety), depression (17% vs. 7%), bipolar disorder (1.6% vs. 0.6%), other psychiatric disorders (3.8% vs. 1.6%), chronic headaches (11% vs. 7%), and pre-injury sleep problems (14% vs. 7%) (*p* < 0.01). Sleep disturbances slightly varied by COI and COI health/environmental and social/economic sub domains, but not by the educational domain, with slightly lower COI scores among those reporting changes to sleep (Table 2).

**Table 2:** Differences in changes to sleep post-concussion by sociodemographic and clinical characteristics.

**Characteristic**	**No, *N* = 1,467^a^**	**Yes, *N* = 3,002^a^**	***p*-value^b^**
Sex			**< 0.001**
Female	622 (42%)	1,755 (58%)	
Male	845 (58%)	1,247 (42%)	
Age at initial visit	14.00 (13.00, 16.00)	15.00 (13.00, 16.00)	**< 0.001**
Race/ethnicity			>0.9
Hispanic	100 (6.8%)	195 (6.5%)	
Non-Hispanic black	169 (12%)	352 (12%)	
Non-Hispanic other	186 (13%)	393 (13%)	
Non-Hispanic white	1,012 (69%)	2,062 (69%)	
Days from injury to initial visit	11 (7, 18)	12 (7, 19)	**< 0.001**
PCSI initial visit	12 (4, 25)	37 (22, 54)	**< 0.001**
Previous concussion	961 (66%)	1,843 (61%)	**0.008**
At least 1 of the selected comorbidities	511 (35%)	1,449 (48%)	**< 0.001**
Medical history: anxiety	227 (15%)	857 (29%)	**< 0.001**
Medical history: ADD/ADHD	236 (16%)	535 (18%)	0.15
Medical history: learning disability	152 (10%)	401 (13%)	**0.004**
Medical history: depression	114 (7.8%)	511 (17%)	**< 0.001**
Medical history: bipolar disorder	9 (0.6%)	47 (1.6%)	**0.007**
Medical history: other psychiatric disorder	23 (1.6%)	115 (3.8%)	**< 0.001**
Medical history: chronic headache	109 (7.4%)	325 (11%)	**< 0.001**
Medical history of sleep problems	103 (7.0%)	420 (14%)	**< 0.001**
COI—overall	90 (72, 96)	89 (69, 96)	**0.020**
COI—educational domain	90 (71, 97)	89 (67, 96)	0.074
COI—health domain	85 (70, 94)	83 (68, 93)	**0.007**
COI—social/environmental domain	87 (70, 95)	86 (66, 94)	**0.015**

^a^n (%); Median (IQR). ^b^Pearson's Chi-squared test; Wilcoxon rank sum test. Bolded values represent statistically significant differences between those reporting sleep disturbance vs. those who did not.

### Multivariate model of sleep disturbance predicting recovery

Table 3 displays the results of the univariate and multivariate models. Factors associated with prolonged recovery in the univariate model included sleep disturbances since injury, sex, age at initial visit, days from injury to initial visit, PCSI at initial visit, history of previous concussion, and medical histories of anxiety, ADD/ADHD, depression, bipolar disorder, chronic headaches and sleep problems. Factors included as potential cofounders in the multivariate logistic regression are displayed in Tables 3, 4.

**Table 3:** Unadjusted and adjusted odds of prolonged recovery by selected sociodemographic and clinical characteristics.

	**Crude**					**Adjusted**				
**Characteristic**	***N***	**Event *N***	**OR^a^**	**95% CI^a^**	***p*-value**	***N***	**Event *N***	**OR^a^**	**95% CI^a^**	***p*-value**
Changes to sleep	4,469	2,553				4,469	2,553			
No			–	–				–	–	
Yes			2.57	2.26, 2.92	**< 0.001**			1.38	1.18, 1.61	**< 0.001**
Sex	4,469	2,553				4,469	2,553			
Male			–	–				–	–	
Female			2.02	1.79, 2.28	**< 0.001**			1.42	1.24, 1.63	**< 0.001**
Age at initial visit	4,469	2,553	1.03	1.01, 1.05	**0.015**	4,469	2,553	1.02	0.99, 1.04	0.3
Race/ethnicity	4,469	2,553								
Hispanic			–	–						
Non-Hispanic black			1.04	0.78, 1.39	0.8					
Non-Hispanic other			1.26	0.95, 1.68	0.10					
Non-Hispanic white			1.06	0.83, 1.35	0.6					
Days from injury to initial visit	4,469	2,553	1.09	1.08, 1.10	**< 0.001**	4,469	2,553	1.09	1.08, 1.10	**< 0.001**
PCSI initial visit	4,469	2,553	1.03	1.03, 1.04	**< 0.001**	4,469	2,553	1.03	1.03, 1.03	**< 0.001**
Previous concussion	4,469	2,553				4,469	2,553			
Yes			–	–				–	–	
No			1.25	1.10, 1.41	**< 0.001**			1.32	1.15, 1.52	**< 0.001**
At least 1 of the selected comorbidities	4,469	2,553				4,469	2,553			
No			–	–				–	–	
Yes			1.35	1.20, 1.53	**< 0.001**			0.83	0.67, 1.02	0.084
Medical history: anxiety	4,469	2,553				4,469	2,553			
No			–	–				–	–	
Yes			1.58	1.37, 1.82	**< 0.001**			1.12	0.89, 1.41	0.3
Medical history: ADD/ADHD	4,469	2,553								
No			–	–						
Yes			1.13	0.96, 1.32	0.14					
Medical history: learning disability	4,469	2,553				4,469	2,553			
No			–	–				–	–	
Yes			1.34	1.12, 1.61	**0.002**			1.48	1.17, 1.87	**0.001**
Medical history: depression	4,469	2,553				4,469	2,553			
No			–	–				–	–	
Yes			1.59	1.33, 1.90	**< 0.001**			0.95	0.74, 1.22	0.7
Medical history: bipolar disorder	4,469	2,553				4,469	2,553			
No			–	–				–	–	
Yes			2.07	1.17, 3.87	**0.017**			0.78	0.40, 1.59	0.5
Medical history: other psychiatric disorder	4,469	2,553								
No			–	–						
Yes			1.25	0.88, 1.78	0.2					
Medical history: chronic headache	4,469	2,553				4,469	2,553			
No			–	–				–	–	
Yes			1.69	1.37, 2.09	**< 0.001**			1.28	0.99, 1.65	0.060
Medical history of sleep problems	4,469	2,553				4,469	2,553			
No			–	–				–	–	
Yes			1.53	1.26, 1.85	**< 0.001**			0.95	0.75, 1.21	0.7
COI—overall	4,469	2,553	1.00	1.00, 1.00	0.7					
COI—educational domain	4,469	2,553	1.00	1.00, 1.00	0.7					
COI—health domain	4,469	2,553	1.00	0.99, 1.00	0.053					
COI—social/environmental domain	4,469	2,553	1.00	1.00, 1.00	0.5					

^a^OR, Odds ratio; CI, Confidence interval. Bolded values represent statistically significant differences between those reporting sleep disturbance vs. those who did not.

**Table 4:** Adjusted odds and standard error of prolonged recovery by selected sociodemographic and clinical characteristics.

**Characteristic**	***N***	**Event *N***	**log(OR)^a^**	**SE^a^**	**95% CI^a^**	***p*-value**
Changes to sleep	4,469	2,553				
No			–	–	–	
Yes			0.32	0.079	0.17, 0.48	**< 0.001**
Sex	4,469	2,553				
Male			–	–	–	
Female			0.35	0.070	0.22, 0.49	**< 0.001**
Age at initial visit	4,469	2,553	0.02	0.014	−0.01, 0.04	0.3
Days from injury to initial visit	4,469	2,553	0.09	0.005	0.08, 0.10	**< 0.001**
PCSI initial visit	4,469	2,553	0.03	0.002	0.03, 0.03	**< 0.001**
Previous concussion	4,469	2,553				
Yes			–	–	–	
No			0.28	0.072	0.14, 0.42	**< 0.001**
Medical history: anxiety	4,469	2,553				
No			–	–	–	
Yes			0.11	0.118	−0.12, 0.34	0.3
Medical history: learning disability	4,469	2,553				
No			–	–	–	
Yes			0.39	0.119	0.16, 0.62	**0.001**
Medical history: depression	4,469	2,553				
No			–	–	–	
Yes			−0.05	0.128	−0.30, 0.20	0.7
Medical history: bipolar disorder	4,469	2,553				
No			–	–	–	
Yes			−0.24	0.349	−0.91, 0.46	0.5
Medical history: chronic headache	4,469	2,553				
No			–	–	–	
Yes			0.25	0.131	−0.01, 0.50	0.060
At least 1 of the selected comorbidities	4,469	2,553				
No			–	–	–	
Yes			−0.19	0.107	−0.40, 0.02	0.084
Medical history of sleep problems	4,469	2,553				
No			–	–	–	
Yes			−0.05	0.122	−0.29, 0.19	0.7

^a^OR, Odds ratio; SE, Standard error; CI, Confidence interval. Bolded values represent statistically significant differences between those reporting sleep disturbance vs. those who did not.

When adjusting for characteristics significantly associated with prolonged recovery in the univariate analysis, patients who reported sleep disturbance since injury had a significantly higher odds of prolonged recovery (OR 1.38, 95% CI 1.18–1.61) in the multivariate model (Tables 3, 4). Patients who had a more days between injury to initial visit (OR 1.09, CI 1.08–1.10) and patients with a higher initial PCSI score (OR 1.03, 95% CI 1.03–1.03) were also associated with higher odds of prolonged recovery. Female patients (OR 1.42, 95% CI 1.24–1.63) and patients without a previous concussion diagnosis (OR 1.32, 95% CI 1.15–1.52) had higher odds of prolonged recovery. A medical history of a learning disability (OR 1.48, 95% CI 1.17–1.87) was significantly associated with prolonged recovery while anxiety, depression, bipolar disorder, chronic headache, and prior sleep problems did not remain associated with prolonged recovery in the multivariate model.

### Moderation of comorbidities on the relationship between sleep disturbance and recovery

Interaction terms examining the moderation effects of individual comorbidities and presence of any comorbidity on the relationship between sleep disturbances and prolonged recovery did not reach statistical significance and were not included in the final model.

## Discussion

This study provides an epidemiological analysis of patient-reported sleep disturbances among a cohort of concussed patients from a large pediatric and adolescent concussion registry. Interestingly, about 2/3 of our patients self-reported changes in sleep, a higher rate than Djukic and colleagues reported (51%; age 3–17) in a systematic review of sleep outcomes after pediatric and adolescent concussion (Djukic et al., 2022). Within the multivariate model, we also found that the presence of sleep disturbances following concussion was the second strongest predictor of prolonged recovery beyond 28 days, only behind female sex. Last, we found that comorbidities did not moderate the relationship between sleep disturbances and prolonged recovery.

There were notable differences in the subjective reporting of sleep disturbances following injury among multiple demographic characteristics and several clinical factors. First, we found sex differences where a higher percentage of females reported sleep disturbance following injury. This is supported by previous research in those 10–35 years of age who were seen within 3 days of injury, which found that after a single concussion, females have significantly increased sleep disturbance (across 4 weeks after injury) while males experience minimal change in sleep (Oyegbile et al., 2017). These findings are reflective of the higher prevalence of girls and women with sleep disorders in the general population, specifically restless leg syndrome (RLS), Rapid Eye Movement (REM) sleep disordered breathing, and insomnia (Mallampalli and Carter, 2014), and likely mirrors concussion reporting behavior where girls and women are more likely to report symptoms (McGroarty et al., 2020). Sex differences in outcomes after concussion may be driven, in part, due to sex differences in sleep, which warrant further investigation. In combination, these results suggest a need for more aggressive surveillance and treatment in young females where the effects of concussion may be more severe and prolonged (Oyegbile et al., 2017). Our research contributes to the growing body of evidence supporting the need for personalized concussion assessments, with careful consideration of factors such as sex, to ensure accurate screening, diagnosis, and treatment.

Second, we found that those who reported sleep disturbances following injury tended to be slightly older. While this finding may give credence to the thinking that younger children may be more resilient to symptom persistence following injury (Ledoux et al., 2019), this may also reflect the fact that older children and adolescents may be more in tune with their body, daily schedule, and sleep need and are better able to describe the presence of sleep disturbances after concussion. Younger children and adolescents may also have more free time to rest or nap compared to their inherently busier older peers.

Interestingly, those who reported sleep disturbance were more likely to present ~1 day later to specialty care compared to their peers without sleep disturbances. This is contrary to what might be expected, where our results show individuals with sleep disturbance have higher overall symptom burden and overlapping symptoms, which one might hypothesize may result in earlier presentation for care. This finding, however, parallels findings among sex differences in concussion and may actually be related to extrinsic factors such as access to care and scheduling availability favoring males (Desai et al., 2019).

Not surprisingly, our analysis demonstrated differences between categories of COI, specifically within the social and environmental domain and overall scores. Specifically, those that reported sleep disturbance following injury had lower social and environmental and overall scores, but not educational, which may represent a difference in housing environment, socioeconomic status, neighborhood noise, and greenspace, among others. These results are clinically important because they highlight the fact that access to care and recovery profiles following a concussion may be confounded by differences in opportunity that are magnified through systemic disparities (Wallace and Mannix, 2021). Thus, we urge clinicians to try to better understand the different environments in which their patients reside and the unique needs of those communities, and to approach clinical management and recommendations with these contexts in mind.

Regarding previous history of concussion, a greater proportion of pediatric and adolescent patients who had no previous concussion reported sleep disturbance compared to those who had a previous injury. There is conflicting data in the literature, however, where others have found that individuals with a history of repeated concussion showed a longer concussion duration and higher reported sleep disturbance following subsequent injury (Oyegbile et al., 2020). This finding may, in part, be a result of the fact that families of individuals sustaining a second concussion may be more familiar with treatment recommendation and may be more apt to begin them earlier, specifically those related to sleep hygiene that were given during care for their previous injury.

PCSI was significantly greater at initial visit for both pediatric and adolescent patients that reported sleep disturbances which may reflect that poor sleep may amplify concussion symptoms. We expected some symptom overlap since those that report sleep disturbance also tend to have co-ocurring symptoms of fatigue, poor concentration, and drowsiness, among others (DuPrey et al., 2022).

Another important finding is the prevalence of a medical history of co-occurring conditions in those reporting subjective sleep disturbance. Previous research from our group has described worsening of pre-existing mood symptoms following concussion (Master et al., 2024), but we did not find a statistical interaction between co-occurring conditions, sleep, and prolonged recovery in the multivariate model. The interaction of these variables *in vivo* is likely complex and individualized.

Finally, we found a difference in prevalence of sleep disturbances between groups according to the length of their clinical course (calculated as number of days from injury to final visit), with individuals that self-reported sleep disturbance after injury taking on average 16 days longer to complete their clinical course of care, which is consistent with previous literature (Bramley et al., 2017). This hypothesis was also supported by our multivariate regression analysis showing a 1.37 OR for recovery past 28 days for those who self-reported sleep disturbance after controlling for other significant variables associated with prolonged recovery. Interestingly, sleep disturbance was the strongest predictor of prolonged recovery, even moreso than other previously described and known clinical contributors to prolonged recovery such as delayed presentation for care, higher symptom burden, and presence of a learning disability. These results support the most recent concussion consensus statement review that describes patients who report sleep disturbance within the first 10 days following sport-related concussion have a higher risk for persistent symptom burden (Patricios et al., 2023), and our study extend this to increased risk for prolonged clinical recovery past 28 days. Our results report on concussions of various mechanisms, not only sport-related, and illuminate an area for improvement in clinical practice for sleep recommendations and early referral. Specifically, our findings highlight a potential interplay between sleep disturbances and sex following concussion in pediatric and adolescent patients.

### Limitations

Data presented is from a specialty care concussion program registry within a single healthcare network in the Northeastern United States and may not be generalizable to other locations or healthcare settings. Additionally, we used a self-report measure of sleep disturbance from prior to injury which could introduce bias. Future studies should aim to incorporate subjective and objective measures of sleep to capture both patient experience and behavior.

## Conclusion

Our study found that the presence of a simple self-reported sleep disturbance at initial visit post-injury was the second strongest predictor of prolonged recovery past 28 days, only behind female sex. Other predictive factors identified in our analyses—later presentation, greater symptoms, concussion history, and learning disability, confirm findings that have also been more frequently highlighted in the literature, while the effect of sex differences and sleep disturbance on concussion recovery appear to be the strongest relationships. These results have important implications for individualizing recovery strategies by adding sleep disturbance as an independent predictor. Specifically, clinicians should be aware of the high rate of sleep disturbance following injury, and the importance of sleep to overall recovery and health-related quality of life while encouraging holistic treatment of the entire individual, including their lifestyle and behaviors, as opposed to simply treating a singular condition.

## Data Availability

The raw data supporting the conclusions of this article will be made available by the authors, without undue reservation.
